# Circulating Galectin-3 and Atrial Fibrillation Recurrence after Catheter Ablation: A Meta-Analysis

**DOI:** 10.1155/2019/4148129

**Published:** 2019-04-02

**Authors:** Guangping Zhang, Yongquan Wu

**Affiliations:** Department of Cardiology, Beijing Anzhen Hospital, Capital Medical University, Beijing, China

## Abstract

**Background:**

Galectin-3 (Gal-3) is involved in fibrosis and heart failure. However, epidemiological studies evaluating the association between Gal-3 and atrial fibrillation (AF) recurrence after catheter ablation showed inconsistent results. We conducted a meta-analysis to comprehensively evaluate the relationship between baseline circulating Gal-3 levels and AF recurrence in patients undergoing catheter ablation.

**Methods:**

Relevant studies were identified by systematically searching the PubMed and Embase databases. A random-effect model was used to synthesize the results. Sensitivity analyses, performed by omitting one study at a time, were used to evaluate the robustness of the results.

**Results:**

Seven prospective cohort studies including 645 AF patients were included. Within a follow-up duration of up to 18 months, 244 patients developed AF recurrence. Pooled results showed that baseline circulating Gal-3 levels were significantly higher in patients with AF recurrence compared to those without (standardized mean difference: 0.74; 95% confidence interval (CI): 0.21 - 1.27; p = 0.007; I^2^ = 89%). Moreover, higher baseline Gal-3 levels were independently associated with a significantly higher risk of AF recurrence after catheter ablation (risk ratio: 1.17 per unit of Gal-3; 95% CI: 1.01 - 1.35; p = 0.03; I^2^ = 40%), which was independent of age, gender, and left atrial dimension. Sensitivity analyses did not significantly affect the results. However, there was a significant publication bias for predicting efficacy of associating preprocedural Gal-3 levels with AF recurrence.

**Conclusions:**

Higher preprocedural Gal-3 levels may be associated with increased risk of AF recurrence in patients undergoing catheter ablation.

## 1. Introduction

Atrial fibrillation (AF) is one of the most common cardiac arrhythmias, and the incidence of AF increases with aging [1]. Clinically, many AF patients are asymptomatic, while some of them may have symptoms of palpitation and dyspnea. More importantly, AF patients have a significantly higher risk for developing heart failure, stroke, and all-cause deaths compared to those without AF [2]. Catheter ablation, including radiofrequency (RF) ablation and cryoablation, has been recommended as an important alternative therapy for patients with symptomatic AF who are resistant to conventional antiarrhythmic drugs, particularly for those patients with paroxysmal AF (PAF) [2, 3]. The primary strategy of AF catheter ablation is to achieve circumferential pulmonary vein isolation (CPVI), thereby terminating the onset of AF via interrupting the electrophysiological basis of AF pathogenesis [4, 5]. However, according to previous reports, the success rate for the treatment of AF after CPVI varies between 50 and 80%, and a subset of patients will develop AF recurrence after catheter ablation [3]. Interestingly, it has been suggested that many patients do not develop AF recurrence even after reconnection of the pulmonary veins [6]. These results suggested that the potential mechanisms underlying AF recurrence after catheter ablation are complex. A better understanding of the clinical factors that predict AF recurrence is of great clinical significance to better manage AF patients after catheter ablation.

Atrial remodeling, characterized by fibrosis, underlies AF pathogenesis [7, 8]. Galectin-3 (Gal-3) is a fibrosis biomarker that is involved in the initiation and progression of many fibrosis related diseases, such as heart failure (HF), live cirrhosis, and lung fibrosis [9, 10]. Recent studies revealed an epidemiological association between circulating Gal-3 levels and the risk of AF incidence in a community-derived population [11, 12], which is biologically plausible since both myocardial fibrosis and HF are primary risk factors for AF [13, 14]. These findings raised the possibility that higher preprocedural circulating Gal-3 levels may be a risk factor for AF recurrence after catheter ablation. However, results of pilot observational studies evaluating the association between preprocedural circulating Gal-3 levels and the risk of AF recurrence after catheter ablation showed inconsistent results [15–21]. Moreover, the sample sizes of these studies were relatively small and were thus underpowered to detect a statistically significant association between baseline Gal-3 levels and AF recurrence. Therefore, in this study, we conducted a meta-analysis of the observational studies to clarify if baseline circulating Gal-3 levels are predictive for AF recurrence and whether preprocedural Gal-3 levels can independently predict AF recurrence after catheter ablation.

## 2. Methods

We conducted this meta-analysis in accordance with the Meta-analysis of Observational Studies in Epidemiology (MOOSE) [22] and the Cochrane's Handbook [23] guidelines.

### 2.1. Database Search

We searched the PubMed and Embase databases using the term “galectin-3”, or “galectin 3”, combined with “atrial fibrillation”. The search was limited to studies in humans and published in English. We also manually searched the reference lists of the related original and review articles for possible studies. The final literature search was performed on July 2, 2018.

### 2.2. Study Selection

The aim of our study was to evaluate the association between baseline circulating Gal-3 levels and AF recurrence after catheter ablation. Therefore, we included observational studies reporting either of the following outcomes: (1) mean differences of circulating Gal-3 levels between patients with or without AF recurrence or (2) multivariable adjusted relative risks of AF recurrence after catheter ablation based on per unit increase of baseline Gal-3 levels. Other inclusion criteria were as follows: (1) adult patients with AF who were scheduled for catheter ablation for the first time; (2) baseline Gal-3 levels measured before ablation; (3) study follow-up duration of at least 6 months; and (4) reporting at least one of the above outcomes. Letters, editorials, studies without controls, baseline circulating Gal-3 levels not reported or measured, or studies that did not report outcomes of interest were excluded. When studies with overlapping patients were found, data from the study with the largest sample size were included.

### 2.3. Data Extraction and Quality Evaluation

Two authors independently performed the literature search, data extraction, and quality assessment according to the predefined inclusion criteria. Discrepancies were resolved by consensus. The extracted data included study design characteristics, patient characteristics (numbers of included AF patients, mean ages, gender, proportions of patients with PAF, and proportions of patients with coronary artery disease (CAD)), details of catheter ablation procedures, methods for assessment of Gal-3 levels, follow-up durations, and detection strategies of AF recurrence. Outcome data, including means and standard deviations (SDs) of baseline circulating Gal-3 levels in patients with and without AF recurrence, as well as the multivariate adjusted risk ratios (RRs) and 95% confidence intervals (CIs) for the incidence of AF recurrence according to the baseline circulating Gal-3 levels, were also recorded. The quality of the included studies was evaluated using the Newcastle-Ottawa Scale (NOS) [24], which judges the quality of each cohort study with regard to three aspects: selection of the study groups, comparability of the groups, and ascertainment of the outcome of interest.

### 2.4. Statistical Analyses

We used the standardized mean difference (SMD) and its 95% CIs to evaluate differences in circulating Gal-3 levels between patients with or without AF recurrence. We used multivariable adjusted RR and 95% CI to evaluate the association between baseline circulating Gal-3 levels and the risk of AF recurrence after catheter ablation. RRs and their corresponding stand errors (SEs) were calculated from 95% CIs or p values and were logarithmically transformed to stabilize variance and normalize distribution. The heterogeneity among the included studies was assessed using the Cochrane's Q test [23] and the I^2^ [25] test. An I^2^ > 50% indicated significant heterogeneity. A random-effect model was applied to synthesize the results because this is a more generalized method that incorporates the heterogeneity of the included studies when combining the results [23]. Sensitivity analyses, performed by removing individual studies one at a time, confirmed the robustness of the results [26]. Potential publication bias was assessed using funnel plot analysis as well as the Egger regression asymmetry test [27]. We also performed the nonparametric “trim-and-fill” procedure [18] to further assess the possible effect of publication bias on the results of our meta-analysis [23]. This method considers the possibility of hypothetical “missing” studies, imputes their HRs, and recalculates a pooled HR that incorporates the hypothetical missing studies as though they actually existed. RevMan (Version 5.1; Cochrane Collaboration, Oxford, UK) and STATA software (Version 12.0; Stata Corporation, College Station, TX) were used for the meta-analysis and statistical analyses.

## 3. Results

### 3.1. Search Results

**Figure 1:**
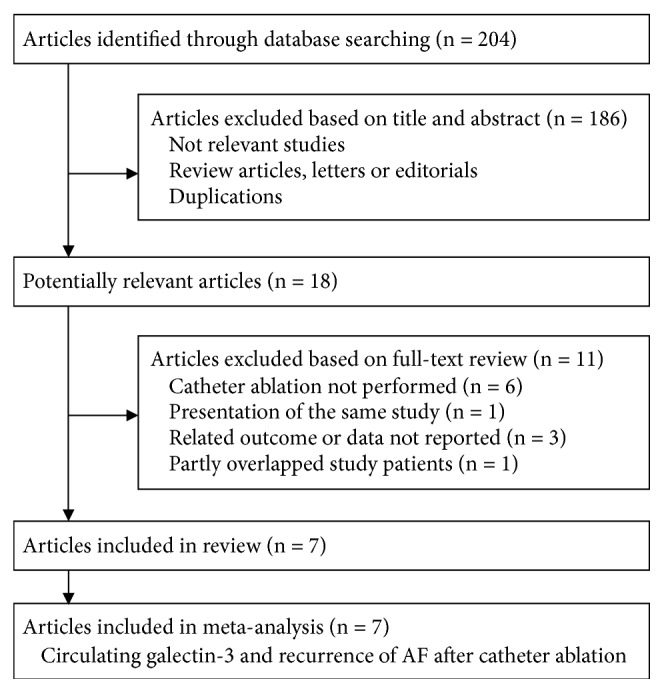
Flowchart of database search.

The literature search process is shown in Figure 1. Briefly, 204 studies were obtained by initial database search and after exclusion of duplicate studies. After screening the titles and abstracts of the publications, 186 studies were subsequently excluded, primarily because they were irrelevant to the objective of the current study. The remaining 18 studies underwent full-text review, and 11 studies were further excluded because six studies did not include AF patients undergoing catheter ablation, three did not report either of the outcomes of interest, one was a duplicate study, and one included an overlapping study population with an already included study. Finally, seven studies [15–21] were included in the current meta-analysis.

### 3.2. Study Characteristics and Quality Evaluation

The characteristics of the included studies are listed in Table 1. Overall, our meta-analysis included seven prospective cohort studies published after 2014 [15–21], with a total of 645 AF patients who underwent catheter ablation. One study included PAF patients exclusively [16] and another included persistent AF patients only [18], while the others included both subtypes of AF [15, 17, 19–21]. The mean ages of the included patients varied between 49 and 63 years, with the percentage of male patients ranging between 44% and 94%. Gal-3 levels were measured with an enzyme linked immunosorbent assay (ELISA) in all of the included studies. As for the ablation strategy, one study applied cryoballoon for CPVI [15], while the others used RF catheter ablation [16–21]. With a mean follow-up of six to 18 months, 244 patients developed AF recurrence as evidenced by Holter examinations. The included studies were generally of good study quality, with the NOS varying between 6 and 9.

### 3.3. Difference of Baseline Gal-3 Levels in Patients with and without AF Recurrence after Catheter Ablation

**Figure 2:**
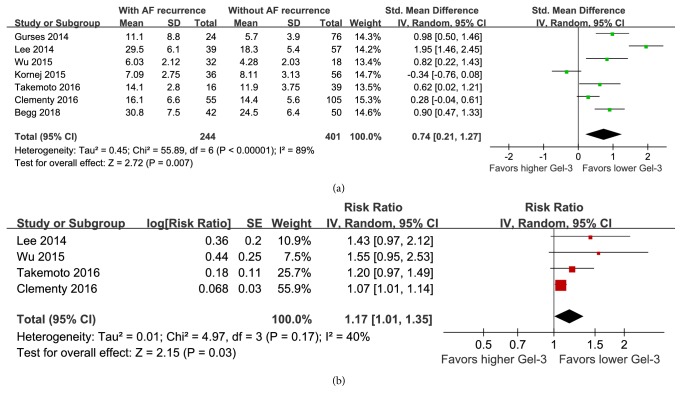
Forest plots showing the association between baseline circulating Gal-3 levels and AF recurrence in patients undergoing catheter ablation. (a) Forest plots for the differences in baseline Gal-3 levels in patients with and without AF recurrence; (b) forest plots for the predictive efficacy of baseline Gal-3 levels for determining the risk of AF recurrence after catheter ablation.

All of the included seven cohort studies reported baseline circulating Gal-3 levels in patients who developed or did not develop AF recurrence after catheter ablation. Pooled results with a random-effect model showed that baseline circulating Gal-3 levels were significantly higher in patients who developed AF recurrence compared to patients who did not develop AF recurrence after ablation (SMD: 0.74; 95% CI: 0.21 to 1.27; p = 0.007; Figure 2(a)) with considerable heterogeneity (p for Cochrane's Q test < 0.001; I^2^ = 89%). Sensitivity analyses did not significantly change the results (SMD: 0.53 - 0.92; p all < 0.05). These results suggest that patients who developed AF recurrence after catheter ablation had higher preprocedural circulating Gal-3 levels compared to those who did not develop AF recurrence.

### 3.4. Predictive Efficacy of Baseline Gal-3 Levels for Determining the Risk of AF Recurrence after Catheter Ablation

Four studies with 361 patients reported the multivariable adjusted association between baseline Gal-3 levels and the risk of AF recurrence after catheter ablation [16, 18–20]. All of the four studies adjusted age, gender, left atrial dimension (LAD), while one of them also adjusted baseline level of N terminal pro B type natriuretic peptide (NT-proBNP) [16]. Pooled results showed that higher baseline Gal-3 levels were independently associated with a significantly higher risk of AF recurrence after catheter ablation (RR: 1.17 per unit of Gal-3; 95% CI: 1.01 to 1.35; p = 0.03; Figure 2(b)) with moderate heterogeneity (p for Cochrane's Q test = 0.17; I^2^ = 40%). Sensitivity analyses did not significantly change the overall results (RR: 1.13 to 1.28; p all < 0.05). These results suggest that higher preprocedural circulating Gal-3 levels may be an independent predictor of AF recurrence in patients undergoing catheter ablation.

### 3.5. Publication Bias

**Figure 3:**
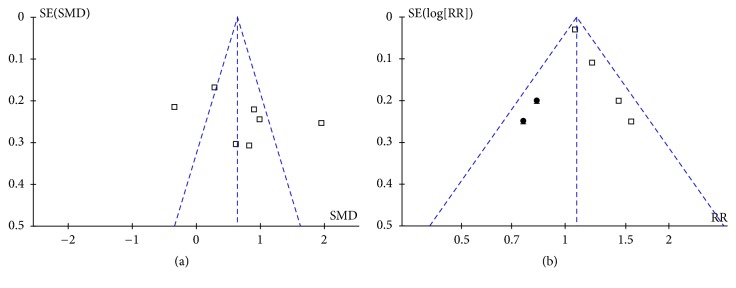
Funnel plots showing estimated publication biases. (a) Funnel plots for the differences in baseline Gal-3 levels in patients with and without AF recurrence; (b) funnel plots with “trim-and-fill” analysis for the predictive efficacy of baseline Gal-3 levels for determining the risk of AF recurrence after catheter ablation. Black dots indicate imputed studies.

**Figure 4:**
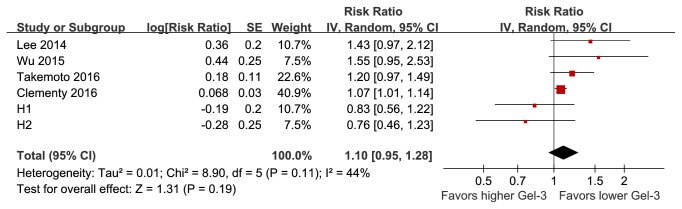
Forest plots showing the predictive efficacy of baseline Gal-3 levels for determining the risk of AF recurrence after catheter ablation after incorporating two imputed studies with “trim-and-fill” analysis.

Publication bias for the current meta-analysis was difficult to estimate because only four to seven studies were included. The funnel plots appeared to be symmetrical upon visual inspection for differences in baseline Gal-3 levels in patients with and without AF recurrence (Figure 3(a)), but not for the association between baseline Gal-3 levels and the risk of AF recurrence (Figure 3(b)). For the latter outcome, including two imputed studies, the “trim-and-fill” method achieved symmetry of the funnel plots. However, the pooled results were insignificant after including these two hypothetical studies (RR: 1.10 per unit of Gal-3; 95% CI: 0.95 to 1.28; p = 0.19; Figure 4). These results suggest that the association between baseline Gal-3 levels and the risk of AF recurrence may be affected by publication bias.

## 4. Discussion

In this meta-analysis we pooled the results of all available prospective cohort studies and found that baseline circulating Gal-3 levels are significantly higher in patients with AF recurrence compared to those without after catheter ablation. Moreover, preprocedural circulating Gal-3 levels are independently associated with a higher risk of AF recurrence after catheter ablation. Specifically, an incremental increase of 1 ng/mL in baseline Gal-3 is associated with a 17% higher risk of AF recurrence, which is independent of age, gender, and baseline LAD of the patients. However, there was publication bias in our analysis regarding the predictive efficacy of baseline Gal-3 levels for determining AF recurrence. Taken together, these results suggest that higher preprocedural circulating Gal-3 levels may be an independent predictor of AF recurrence for patients undergoing catheter ablation.

Gal-3 is a member of the beta-galactoside-binding proteins, which are overproduced and released in pathophysiological conditions related to inflammation and fibrosis [28]. Through induction and activation of tumor growth factor beta (TGF-beta) and Smad3, Gal-3 is released by macrophages and triggers various remodeling related signaling pathways that contribute to the pathogenesis of many fibrosis related diseases, including HF [29]. Indeed, in a previous experimental study, overexpression of Gal-3 in the myocardium promoted infiltration of macrophages and mast cells, resulting in myocardial fibrosis and hypertrophy [30]. As a disease associated with inflammation and fibrosis [7, 31], AF pathogenesis and progression also likely involve Gal-3. Pilot studies demonstrated that circulating Gal-3 levels were significantly elevated in AF patients compared to controls and were higher in patients with persistent AF compared to those with PAF [32]. Subsequent cohort studies indicated that higher Gal-3 levels may predict AF incidence in the general population [11, 12], although the association between higher Gal-3 levels and increased risk of AF incidence may be explained by conventional AF risk factors [33]. Further studies using echocardiography showed that circulating Gal-3 levels significantly correlated with left atrial volume index in AF patients with preserved left ventricular function, suggesting a direct relationship between circulating Gal-3 levels and extent of atrial remodeling [34]. This association was further confirmed in another study using delayed enhancement magnetic resonance imaging, which demonstrated an atrial electromechanical delay in PAF patients [35]. Moreover, recent studies showed that increased Gal-3 levels correlated with increased thrombogenicity in persistent AF patients as reflected by reduced LA appendage flow velocity, appendage remodeling, and thrombus formation in transesophageal echocardiography [36, 37]. These findings suggested that baseline Gal-3 levels reflect the risk of stroke in AF patients. Our study expanded the above findings by showing that patients with AF recurrence after catheter ablation have higher baseline Gal-3 levels and that higher preprocedural circulating Gal-3 levels may be an independent predictor of AF recurrence for patients undergoing catheter ablation. Our results demonstrate that, despite the role of Gal-3 in AF pathogenesis, baseline circulating Gal-3 levels may predict the response to catheter ablation in AF patients. This is consistent with the results of a previous study that showed that baseline Gal-3 levels independently predict outcomes in patients with reduced left ventricular systolic function attributed to ablation of persistent AF [38]. Taken together, Gal-3 is likely involved in AF pathogenesis and progression, risk stratification for stroke incidence, and treatment response to catheter ablation. Further studies are needed to extensively understand the role of Gal-3 in AF. Also, targeting galectin-3 related inflammation and fibrosis process, perhaps via statins, may be a potential strategy to prevent AF recurrence after catheter ablation. Further studies are warranted.

There are some limitations in our meta-analysis that should be considered when interpreting our results. Firstly, we only included seven cohorts, and we did not have access to individual patient-based data, which prevented us from performing stratified analyses to elucidate the association between baseline Gal-3 levels and risk of AF recurrence in patients with different clinical characteristics, such as those with PAF or persistent AF. Secondly, although multivariable adjusted RR was extracted for the meta-analysis, we could not fully exclude the chance that some residual factors remained that may have confounded the association between Gal-3 levels and the risk of AF recurrence. In fact, the correlations of these factors, such as left atrial size, duration of AF, left ventricular ejection fraction, presence of heart failure, BMI, and medication used (including stains) with galectin-3 may confound the potential association between galectin-3 and AF recurrence. However, since these factors were not statistically significant in univariate analysis for the potential predicting of AF recurrence in the original studies, they were not finally incorporated into the multivariate models. We acknowledged this as an important limitation of our study. Studies with adequate statistical power to incorporate these factors in the multivariate analyses are warranted. Thirdly, a causal relationship between higher baseline Gal-3 levels and the risk of AF recurrence could not be concluded based on the findings of our study because only observational studies were included in our meta-analysis. Fourthly, as mentioned above, potential publication bias may have influenced the reliability of our findings regarding the association between higher baseline Gal-3 levels and the risk of AF recurrence. In addition, it remains unknown whether the associations between galectin-3 and AF recurrence are different in RF and cryoablation, because studies regarding the role of galectin-3 in AF patients with cryoablation are rare. This has been also listed as a limitation. Finally, AF recurrence after catheter ablation is likely multifactorial. Accordingly, predictive models based on multivariate analyses may be more efficient. It remains unclear if adding baseline Gal-3 levels into these models for AF recurrence could improve the predictive efficacies.

In conclusion, our meta-analysis indicated that patients with AF recurrence after catheter ablation had higher baseline Gal-3 levels and that higher preprocedural circulating Gal-3 levels are an independent predictor of AF recurrence for patients undergoing catheter ablation.

## Figures and Tables

**Table 1:** Characteristics of the included studies.

Author (year)	Country	Patient number	PAF	Mean age	Male	CAD	Gal-3 method	CA details	Number of cases with AF recurrence	Mean follow-up	Outcomes reported	Variables adjusted	NOS
			%	years	%	%				months			
Gurses (2014)	Turkey	100	NR	57	44	0	ELISA	CB-CPVI	24	12	Baseline difference of Gal-3	NA	6 (3/2/1)
Lee (2014)	China	96	100	NR	NR	NR	ELISA	RF-CPVI	39	18	Baseline difference of Gal-3 and risk estimation for AF recurrence	Age, gender, LAD, and NT-proBNP	6 (3/2/1)
Wu (2015)	China	50	0	49	94	0	ELISA	RF-CPVI Plus	32	12	Baseline difference of Gal-3 and risk estimation for AF recurrence	Age, gender, and LAD	9 (4/3/2)
Kornej (2015)	Germany	92	51	62	65	NR	ELISA	RF-CPVI Plus	36	6	Baseline difference of Gal-3	NA	8 (4/2/2)
Takemoto (2016)	US	55	53	63	82	15	ELISA	RF-CPVI Plus	16	12	Baseline difference of Gal-3 and risk estimation for AF recurrence	Age, gender, type of AF, and LAD	9 (4/3/2)
Clementy (2016)	France	160	55	61	71	NR	ELISA	RF-CPVI Plus	55	12	Baseline difference of Gal-3 and risk estimation for AF recurrence	Age, gender, and LAD	9 (4/3/2)
Begg (2018)	UK	92	67	58	60	5	ELISA	RF-CPVI Plus	42	12	Baseline difference of Gal-3	NA	9 (4/3/2)

AF, atrial fibrillation; PC, prospective cohort; PAF, paroxysmal AF; NR, not reported; Gal-3, galectin-3; CAD, coronary artery disease; CA, catheter ablation; CB, cryoballoon; RF, radiofrequency; CPVI, circumferential pulmonary vein isolation; CPVI plus, includes CPVI with one or more of adjuvant ablations in cavotricuspid isthmus, mitral isthmus, left atrial roof, the basal posterior wall, superior vena cava or complex fractionate atrial electrograms; ELISA, enzyme linked immunosorbent assay; LAD, left atrial dimension; NT-proBNP, N terminal pro B type natriuretic peptide; NOS, Newcastle-Ottawa Scale.
